# Percutaneous access of an intraoperatively recanalized mid-superficial femoral artery for distal lower limb revascularization

**DOI:** 10.1016/j.jvscit.2023.101170

**Published:** 2023-03-30

**Authors:** Angelos Karelis, Doriana Ferrara, Björn Sonesson, Nuno Dias

**Affiliations:** a Vascular Center, Department of Thoracic and Vascular Diseases, Skåne University Hospital, Malmö, Sweden; b Department of Clinical Sciences Malmö, Lund University, Malmö, Sweden; c ASST Fatebenefratelli Sacco, AOU Federico II, Milan, Italy

**Keywords:** Advanced endovascular techniques, Chronic limb threatening ischemia, Peripheral arterial disease, Thigh puncture

## Abstract

A 61-year-old male patient presented with rest pain and ulceration in his left leg 1 week after a hybrid procedure with bilateral external iliac stenting, common femoral artery thromboendarterectomy, and left-sided femoral popliteal bypass with an in situ saphenous vein. The bypass had been stented intraoperatively but had again become occluded directly after surgery. In the present report, we demonstrate the usefulness of direct percutaneous access to the mid-superficial femoral artery that had been intraoperatively recanalized via brachial artery access during the same procedure. This innovative combination of approaches allows for proximal and distal lower limb revascularization with stenting when avoidance of femoral artery access is considered appropriate.

The treatment of patients with chronic limb threatening ischemia (CLTI) is demanding, because of the complexity of the lesions, patients often have several comorbidities, and the high related mortality. Even if it is advisable to treat long lesions with an adequate vein for autologous bypass with open surgery in light of the recently reported BEST-CLI (best endovascular vs best surgical therapy in patients with critical limb ischemia) study,^1^ the endovascular strategy is still commonly used to treat CLTI patients, including those with TASC (TransAtlantic InterSociety Consensus) C/D lesions,^2^ owing to the advances in materials and techniques. Cases for which either open or endovascular revascularization fails are technically demanding and the need for new access sites could arise. Even if in recent years platforms for upper extremity access in lower limb revascularization have been developed, at times, these will not be applicable or available.

## Surgical technique

We describe the case of a 61-year-old male patient with a history of hypertension and smoking, who was admitted to our department for progression of rest pain and ulceration in his left leg. Only 1 week before, he had undergone a hybrid procedure with bilateral external iliac stenting, bilateral common femoral artery (CFA) thromboendarterectomy, and left-sided femoropopliteal bypass with an in situ saphenous vein. The bypass had poor flow intraoperatively and was stented distally to shorten an already long procedure. However, it became occluded again directly after surgery. His symptoms progressed, and computed tomography angiography confirmed reocclusion of the bypass, flush occlusion of the superficial femoral artery (SFA), and a short, significant stenosis at the deep femoral artery distal to the thromboendarterectomy segment on the left side. Subacute revascularization of the SFA was considered necessary for limb salvage.

Because his recent surgery had resulted in inguinal hematomas, percutaneous femoral access could not be achieved. Reexploration of the groin was considered to be associated with a high risk of infection at the same time that on-line flow to the ulcer was needed.^3^ With local anesthesia, the right brachial artery (BA) was punctured using an 18-guage needle, and a 5F sheath 100 cm long was positioned. Next, 100 IU/kg of intra-arterial heparin was administered as a bolus and reinforced hourly to maintain the activated clotting time at >250 seconds. Digital subtraction angiography showed occlusion of the SFA from its origin to the P3 segment of the popliteal artery (PA), severe stenosis of the distal profunda femoral artery, and heavily calcified, but still patent, tibial vessels.

To maximize inflow before SFA recanalization, the deep femoral artery was stented (EverFlex Entrust 6/20 mm; Medtronic Vascular, Santa Rosa, CA) with good technical results through the brachial access. Percutaneous antegrade access into the proximal SFA failed.

**Fig 1:**
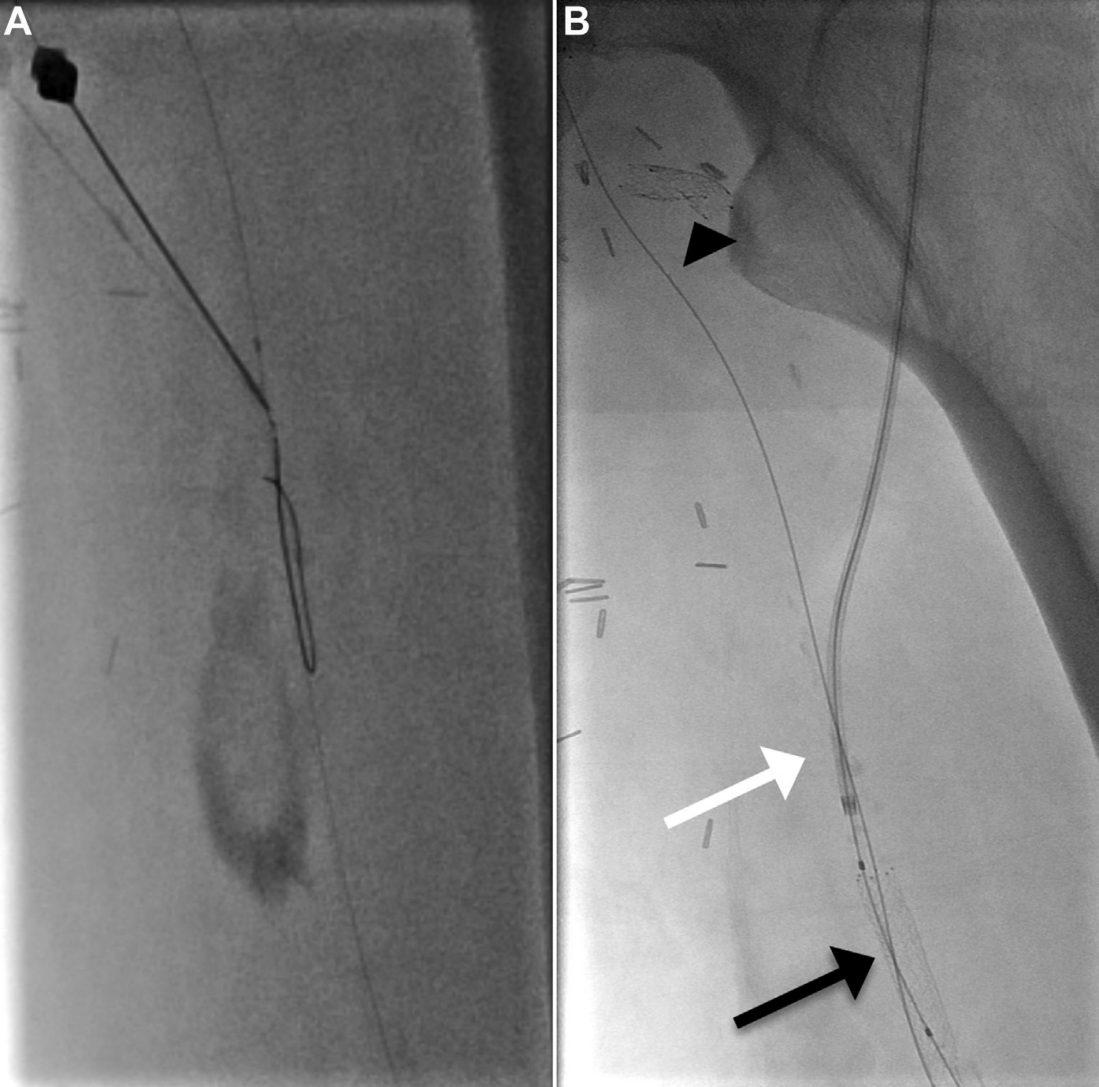
**A,** Percutaneous puncture of an intraoperatively subintimally recanalized superficial femoral artery (SFA) from brachial artery (BA) access. SFA access was ∼15 cm from the origin. **B,** A 4F, 23-cm-long sheath was positioned (*white arrow*), and the distal stents were released through this access (*black arrow*). Hemostasis was achieved with a balloon (*arrowhead*) that was inflated while removing the introducer.

**Fig 2:**
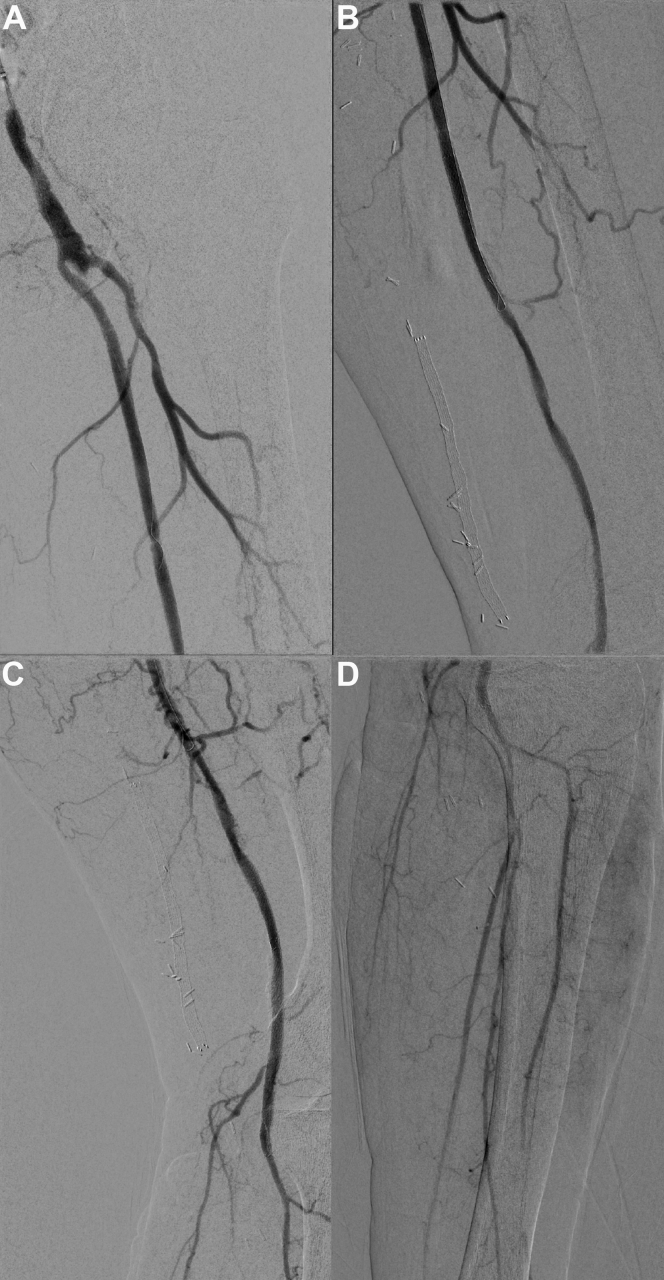
Completion angiogram after revascularization through a combined brachial artery (BA) and superficial femoral artery (SFA) approach showing a patent SFA and deep femoral artery **(A** and **B)** and patent popliteal artery (PA) and tibioperoneal trunk **(C** and **D)** with no signs of dissection or distal embolization.

Retrograde access was performed from the dorsal pedal artery under ultrasound guidance, with an 18-gauge needle. Intraluminal recanalization of the femoropopliteal segment was not possible; thus, a subintimal channel from the P2 segment of the PA to the CFA was created using a 0.018-in. guide wire (Terumo GA1830 Glidewire Advantage; Terumo, Tokyo, Japan) and a support catheter (CXI; Cook Medical Inc, Bloomington, IN). Reentry in the true lumen was achieved at the SFA origin level, and the guide wire from retrograde access was snared from the BA to establish a through-and-through system. Predilation of the subintimal channel was performed from the BA access with 3-mm and 5-mm diameter balloons. Stenting of the mid- and distal SFA was not possible from the BA owing to the insufficient shaft length of the available stents (135 cm). Consequently, the SFA was punctured antegradely directly after its recanalization ∼15 cm from the origin, under fluoroscopic guidance, to enter the subintimal channel previously created (Fig 1). A 4F, 23-cm-long sheath was positioned (Prelude; Merit Medical Systems, South Jordan, UT), and the wire could be passed into the tibial artery. The BA wire was pulled back into the subintimal channel proximal to the SFA puncture, and the pedal access was removed, with manual compression used to achieve hemostasis. The distal SFA and proximal PA segments were dilated with 6- and 5-mm balloons, respectively, and two Pulsar-18 stents (Biotronik AG, Bülach, Switzerland) were placed antegradely from the thigh access (5 mm distally and 6 mm proximally). The BA wire was now advanced into the SFA stents. The 4F sheath to the SFA was then removed while performing hemostasis by inflating a 6-mm balloon for 5 minutes at the puncture site as an endoclamp catheter. Hemostasis was confirmed angiographically after removal of the balloon. Finally, the procedure was completed by placing and dilating a stent in the very proximal part of the SFA, which was easily reachable from the BA access (6-mm Pulsar-18 stent; Biotronik AG). The completion angiogram showed patent SFA, PA, and deep femoral arteries without any dissection or significant stenosis (Fig 2). Manual compression was applied to the BA. The toe brachial index increased from 0.17 to 0.60 postoperatively with immediate relief from rest pain. Duplex ultrasound confirmed the patency of the revascularization without significant stenosis and no signs of pseudoaneurysm at the puncture sites. The patient had an uneventful postoperative course and was discharged on postoperative day 13 after surgical revision of his foot ulcers with a prescription for double antiplatelet therapy for 3 months. At the 1-, 3-, and 6-month follow-up examination with duplex ultrasound, the reconstruction was patent with no signs of significant stenosis. The patient provided written informed consent for the report of his case details and imaging studies.

## Discussion

To the best of our knowledge, this is the first description of antegrade puncture of an intraoperatively recanalized SFA in the thigh. Gutzeit et al^4^ presented a series of 100 consecutive patients revascularized through antegrade proximal SFA access. However, in their report, the SFA was patent at this level. The results showed that the incidence rate of pseudoaneurysms was comparable to CFA retrograde access reported in a similar series (10.2% vs 7.7%).^5^ This similarity was also reported for inguinal hematomas. Moreover, the use of vascular closing devices seems to minimize the occurrence of inguinal complications compared with manual compression in proximal SFA antegrade access.^6^ These studies investigated only antegrade access to the very proximal SFA, and the more distal SFA access, such as performed in our patient, was not used. In our experience, distal puncture of the SFA has been easy to achieve, for both the presence of the guide wire and the previous dilatation of the subintimal space. More importantly, the antegrade direction does not seem to risk hemostasis with the balloon, which has been previously described in the same location when retrogrades punctures were performed.^7^

The present case had additional complexity because antegrade proximal entry into the SFA was not feasible owing to the absence of an adequate SFA stump. Retrograde access from the foot was preferred to access via the PA. Retrograde PA access can be performed if the P1 segment is patent, because the P2 and P3 segments are closer to the popliteal vein and, thus, more exposed to iatrogenic arteriovenous fistula creation.^8^^,^^9^ Furthermore, this access could be difficult in extremely calcified arteries, and a prone patient position is needed, with the disadvantages of not permitting an easy through-and-through technique and being a time-consuming strategy.

When it is not possible to revascularize the SFA with the traditional ipsilateral or contralateral technique, BA access at the antecubital fossa can be used. Although it might seem convenient, this antegrade approach does not offer the same support to sheaths, catheters, and guide wires. The same issues also occur with the increasingly popular radial artery access. More importantly, the shaft length will often be insufficient to reach the distal SFA and PAs, particularly in tall patients. In such cases, this new alternative could prove useful when distal retrograde access is considered risky to the outflow.

## Conclusions

The described technique of direct antegrade access into a freshly intraoperatively recanalized SFA offers a feasible and safe method to treat CLTI patients with femoropopliteal segment occlusions when proximal femoral artery access is limited. It allows for access to the distal arteries but must be combined with BA or retrograde access to treat the segment proximal to the puncture site and to achieve hemostasis.
